# Maintaining intraoperative normothermia reduces blood loss in patients undergoing major operations: a pilot randomized controlled clinical trial

**DOI:** 10.1186/s12871-018-0582-9

**Published:** 2018-09-08

**Authors:** Jie Yi, Hao Liang, Ruiyue Song, Hailu Xia, Yuguang Huang

**Affiliations:** Department of Anesthesia, Peking Union Medical College Hospital, Chinese Academy of Medical Science, 1 Shuaifuyuan, Dongcheng District, Beijing, 100730 China

**Keywords:** Active forced warming, Passive warming, Intraoperative bleeding, Major surgery, Inadvertent intraoperative hypothermia

## Abstract

**Background:**

Inadvertent intraoperative hypothermia (core temperature < 36 °C) is a common but preventable adverse event. This study aimed to determine whether active intraoperative warming reduced bleeding in patients undergoing major operations: open thoracic surgery and hip replacement surgery.

**Methods**/**Design:**

The study was a pilot, prospective, parallel two-arm randomized controlled trial. Eligible patients were randomly allocated to two groups: passive warming (PW), with application of a cotton blanket (thermal insulation), or active warming (AW), with a forced-air warming system. The primary endpoint was intraoperative blood loss, and secondary endpoints were surgical-site infection, cardiovascular events, and length of stay in the post-anesthesia care unit, intensive care unit, and hospital.

**Results:**

Sixty-two patients were enrolled. Forced-air active warming maintained intraoperative normothermia in all AW subjects, whereas intraoperative hypothermia occurred in 21/32 (71.8%) of PW patients (*p* = 0.000). The volume of blood loss was more in the PW group (682 ± 426 ml) than in the AW group (464 ± 324 ml) (*p* < 0.021), and the perioperative hemoglobin value declined more in the PW group (28.6 ± 17.5 g/L) than in the AW group (21.0 ± 9.9 g/L) (*p* = 0.045). However, there were no difference in other clinical outcomes between two groups.

**Conclusion:**

Intraoperative active warming is associated with less blood loss than passive warming in open thoracic and hip replacement operations in this pilot study.

**Trial registration:**

This trial was registered with Clinicaltrials.gov (Identifier: NCT02214524) on 27 August 2014.

## Background

Inadvertent perioperative hypothermia, defined as core temperature below 36 °C during operation, occurs commonly in patients undergoing general or regional anesthesia when their core body temperature is not regulated [1]. Core body temperature declines by as much as 1.6 °C within the first hour after the induction of anesthesia, which increases the risks of inadvertent hypothermia [2], mortality, blood loss/blood transfusion [3–6], longer hospital stay [7], and surgical-site infection [8, 9].

Inadvertent hypothermia has been associated with intraoperative bleeding, but the relevant published data are inconclusive. Schmid et al. [4] evaluated blood loss and transfusion requirements in 60 patients undergoing primary total hip arthroplasties, who were randomly assigned to normothermia or mild hypothermia; intra- and post-operative blood loss were found significantly more in the hypothermic patients than in the normothermic patients, and a typical decrease in core temperature resulted in about 500 mL blood loss [4]. Winkler et al. [3] aggressively warmed patients to maintain a tympanic-membrane temperature of 36.5 °C compared to core temperature of 36.0 °C in patients receiving the conventional warming; they found that intraoperative blood loss was significantly more in the latter group. Besides increasing blood loss [10, 11], hypothermia can impair blood coagulation, as measured with activated partial thromboplastin time and bleeding time [12]. In a meta-analysis of 14 published randomized clinical trials comparing blood loss in normothermic and mildly hypothermic (34-36 °C) surgical patients, mild hypothermia (< 1 °C) was associated with significantly increased blood loss of about 16% (4–26%) and increased relative risk for transfusion of about 22% (3–37%) [5]. Although the above-cited authors have concluded that maintaining perioperative normothermia reduces blood loss and transfusion requirements [13] other data are not supportive [14, 15].

This study was part of a national perioperative health-care quality improvement and health economic research program in China. Limited data are available in China on whether AW reduces bleeding in patients undergoing major open operations. Thus, this pilot study was undertaken to evaluate objectively this issue in patients undergoing open thoracic or hip replacement surgery.

## Methods

### Ethical status

This study protocol was approved by the Institute Review Boards of Peking Union Medical College Hospital in Beijing, China.

### Study design

This is a pilot prospective, parallel two-arm randomized controlled trial that is registered with clinicaltrials.gov (identifier: NCT02214524) in 2014. Due to the nature of intervention, it is an open-label study. Eligible subjects were those undergoing either initial unilateral total hip replacement or open thoracic operations (pulmonary lobectomy or esophagectomy). Inclusion criteria were: age18 to 80 years and American Society of Anesthesiologists Physical Status 1–3. Exclusion criteria were: history of excessive bleeding; partial thromboplastin time > 35 s; prothrombin time > 35 s; fibrinogen < 200 mg/dL; platelet count < 100,000/L; history of infection and fever within 4 weeks before surgery; use of steroid or immunosuppressant within 4 weeks before surgery; Raynaud’s disease; hypothyroidism or hyperthyroidism; Uncontrolled insulin-dependent diabetes mellitus (preoperative glucose > 250 mg/dL); preoperative temperature above 37.5 °C or less than 36 °C.

Patients eligible by the above criteria gave written, informed consent for participation in this study. Patients were enrolled and randomly allocated to either a passive warming (PW) group or an active warming (AW) group. Randomized numbers were generated by SAS 9.0 (SAS Institute, Cary, NC) and sealed in envelopes.

#### PW group

Patients were covered with unwarmed cotton blanket (thermal insulation) throughout the operation, from the preoperative holding area, operating room, to post-anesthesia care unit (PACU). Cover with unwarmed cotton blanket, usually one layer, is currently a routine practice in most institutions in China and can be considered standard of care.

#### AW group

Patients were covered with forced-air blankets connected to a warming unit (Bair-Hugger Patient Warmer, 3 M, St. Paul MN, USA), which has been reported to be the most effective warming device [16]. The blankets were placed on the operating room table and current turned on before the patient arrived. This protocol allowed the care provider to focus on the patient and be prepared from the start of the procedure. All patients were pre-warmed for 15–30 min with an upper-body blanket in the holding area and then moved onto the operating table prior to induction, where a pre-warmed lower-body blanket was already placed. The Bair-Hugger warming unit was set on its highest temperature (43 °C). If patient core temperature was below 37.5 °C, the warming unit was placed on its highest temperature setting or switched to the appropriate setting at the discretion of investigators to maintain normothermia. This program was begun when patients arrived in the operating room waiting area and was terminated at the end of operation, when patients left the operating room and were transferred to the PACU or ICU.

#### All patients received general anesthesia according to the following standard protocol

The regimens were propofol (2–2.5 mg/kg), fentanyl (2–4 μg/kg) and rocuronium (0.8–1 mg/kg) at induction, and sevoflurane (1.5–2 vol%) mixed with O_2_/N_2_O (50%/50%) for maintenance. The core temperature was measured with an infrared tympanic-membrane thermometer (ThermoScan PRO-4000, Braun GmbH, Kronberg, Germany). To avoid bias, the thermometer was calibrated and validated according to the manufacturer’s manual before use. The core temperature was recorded every 15 min from the time the patient was placed in the operating room holding area until he/she was transported to the PACU after surgery. Intraoperative hypothermia was defined as core temperature below 36 °C at any point in the perioperative period. All patients were followed for 30 days after surgery, especially for surgical-site infection. Investigators or study coordinators telephoned the patients or his/her family members if the patients had been already discharged from the hospital.

Primary and secondary endpoints, adverse events, and severe adverse events were collected for analysis.

### Endpoints

The primary endpoint was intraoperative blood loss. Intraoperative blood loss was estimated as the combined change in sponge weights (the density of a sponge is 1 g/ml) and volume of fluid in the suction reservoir, recorded by circulating nurses who were blinded to patients’ core temperatures but not to whether the patients received AW. The need for blood transfusion was determined at physician discretion. The total volume of blood transfused included autologous transfusion and allogeneic transfusion, and the total volume was measured according to volume infused intravenously.

Secondary endpoints were postoperative surgical-site infection; cardiovascular events; length of stay (LOS) in the PACU, ICU and hospital; and shivering. The incidence and intensity of shivering were graded by the scale described by Crossley and Mahajan [17]: 0, no shivering; 1, no visible muscle activity, but piloerection, peripheral vasoconstriction or both present (other causes excluded); 2, muscular activity in only one muscle group; 3, moderate muscular activity in more than one muscle group but no generalized shaking; and 4, violent muscular activity involving the whole body.

### Statistical analysis

Sample-size estimation was based on reference to published articles [4, 5, 15]. As this is a pilot study, we planned to study a total of 60 subjects (30 per group) and then determine the sample size for a definitive study in the future.

Analyses were performed based on the intention-to-treat population, which included all patients except those who failed in the screening. Categorical data were analyzed with Chi-square or Fisher’s exact test. Continuous variables were analyzed using unpaired, two-tailed t-tests or non-parametric analysis. *p* value of less than 0.05 was considered to indicate a statistically significant difference. The data analysis was conducted with SAS 9.0 (SAS Institute, Cary, NC).

## Results

### Baseline characteristics

**Fig. 1:**
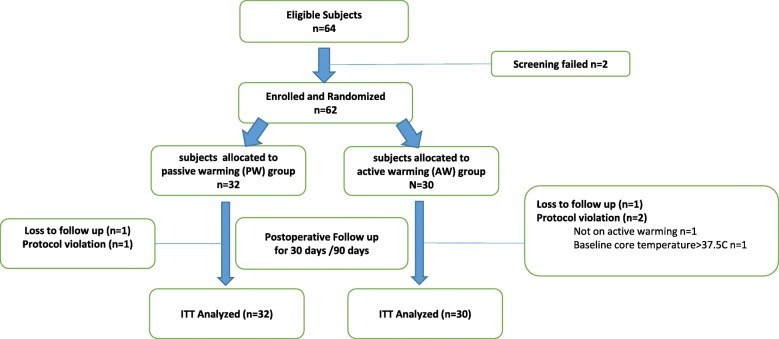
Flow Chart of Study**.** A total of 64 patients were screened. Two patients were excluded; 62 were enrolled and randomly allocated to two groups: passive warming (PW) and active warming (AW). Data analysis was based on the intent-to-treat (ITT) population

Sixty-four patients were screened with the inclusion and exclusion criteria after giving informed consent; 62 patients were eligible. Eligible patients were randomly allocated to a PW or AW group (Fig. 1). Table 1 depicts the baseline characteristics of patients enrolled. The two populations were similar in sex, age, body mass index, smoking history, past medical histories, type of operation, preoperative core temperature, and American Society of Anesthesiologists classification. Hemoglobin, platelet count, international normalized ratio, and fibrinogen values also were similar in the two groups.

**Table 1:** Baseline Characteristics of Patients in Two Study Groups

Varables	Passive Warming Group (*n* = 32)	Active Warming Group (*n* = 30)
Male, *N* (%)	25 (78.1)	21 (70.0)
Age, mean ± std (year)	58.5 ± 11.5	57.9 ± 11.8
BMI, mean ± std	24.7 ± 3.8	23.6 ± 3.8
History of smoking, *N* (%)	17 (53.1)	13 (43.3)
History of Alcholol, *N* (%)	16 (50.0)	17 (56.7)
Past medical history		
Cardiovascular disease, *N* (%)	14 (43.7)	11 (36.7)
Celebrovascular disease, *N* (%)	3 (9.3)	0 (0.0)
Liver disease, *N* (%)	3 (9.3)	3 (10.0)
Kidney disease, *N* (%)	2 (6.3)	0 (0.0)
Diabetes, *N* (%)	5 (15.7)	4 (13.3)
Type of surgery		
Hip replacement, *N* (%)	12 (38.7)	12 (40.0)
Thoracic Surgery, *N* (%)	18 (56.3)	20 (66.7)
Preoperative core temperature, mean ± std (°C)	37.2 ± 0.3	37.3 ± 0.3
ASA grade, *N* (%)		
Class 1	8 (25.0)	8 (26.7)
Class 2	22 (68.7)	21 (70.0)
Class 3	2 (6.3)	1 (3.3)
Hemoglobin, mean ± std, (g/L)	143.8 ± 15.4	137.4 ± 16.5

### Intraoperative characteristics of the two patient groups

Table 2 lists the intraoperative characteristics of the two patient groups, including data on the primary endpoint (intraoperative blood loss). The forced-air based active warming system maintained intraoperative normothermia in all AW patients (0% incidence of hypothermia) for the duration of operations. In contrast, the incidence of intraoperative hypothermia of ≤36.0 °C was 71.8% and of ≤35.5 °C was 37.5% in PW patients, and temperature reached ≤35 °C in three (9.4%) PW patients.

**Table 2:** Intraoperative Characteristics of Patients in Two Study Groups

Variable Name	Passive Warming Group (*n* = 32)	Active Warming Group (*n* = 30)	*p* Value
Core temperature (tympanic membranes)			
< 36.0^0^C, N (%) < 36.0^0^C, *N* (%)	23 (71.8)	0 (0.0)	0.0000
< 35.5^0^C, N (%) < 35.5^0^C, *N* (%)	12 (37.5)	0 (0.0)	0.0001
< 35.0^0^C, N (%) < 35.0^0^C, *N* (%)	3 (9.4)	0 (0.0)	0.2384
Blood loss, mean ± std (ml)			
Total blood loss	681.5 ± 425.8	464.0 ± 324.1	0.0207
Hip repacement	1035.4 ± 491.4	713.5 ± 341.4	
Thoracic surgery	469.2 ± 171.7	311.5 ± 199.0	
Blood transfusion			
Transfused patient, *N* (%)	11 (34.4)	9 (30.0)	0.7124
Volume, mean ± std (ml)	223.3 ± 351.0	137.1 ± 293.9	0.3008
Reduction of hemoglobin, mean ± std (g/L)	28.6 ± 17.5	21.0 ± 9.9	0.0452
Platelet			
Preoperative, mean ± std (10^9^/L)	210.6 ± 59.9	212.8 ± 40.0	0.8699
Postoperative, mean ± std (10^9^/L)	177.9 ± 50.6	177.8 ± 40.4	0.8839
Prothromin Time (PT)			
Preoperative, mean ± std (s)	11.6 ± 0.88	11.3 ± 0.58	0.0439
Postoperative, mean ± std (s)	12.7 ± 0.9	12.6 ± 0.6	0.4158
International Normalized Ratio (INR)			
Preoperative, mean ± std	1.0 ± 0.1	1.0 ± 0.1	0.0623
Postoperative, mean ± std	1.1 ± 0.1	1.0 ± 0.1	0.0815
Fibrinogen			
Preoperative, mean ± std (g/L)	3.4 ± 0.8	3.2 ± 0.9	0.3367
Postoperative, mean ± std (g/L)	3.3 ± 0.8	3.8 ± 1.4	0.1247
Duration of surgery, mean ± std (min)	301.7 ± 98.4	248.3 ± 118.8	0.0579
Ambient temperature, mean ± std (°C)	21.1 ± 1.2	21.1 ± 1.2	0.9655
Intravenous fluid, mean ± std (ml)	2883.1 ± 1037.1	2652.3 ± 1048.7	0.3872
Urine output, mean ± std (ml)	948.4 ± 827.8	587.9 ± 603.8	0.0603
Irrigation fluid, mean ± std (ml)	3270.3 ± 1187.4	2898.2 ± 1315.9	0.2542

The combined volume of intraoperative blood loss for the two operations (hip replacement and thoracic surgery) was significantly less in the AW group than in the PW group (464 ± 324 ml vs 682 ± 423 ml; *p* = 0.0207). Accordingly, hemoglobin values declined less from preoperative values to postoperative values in the AW group (21.0 ± 9.9 g/L) than in the PW group (28.6 ± 17.5 g/L) (*p* = 0.0452). The proportion of patients that received blood transfusion in the PW group (11/32; 34.4%) and the AW group (9/30; 30.0%) were similar. The volume of blood transfused was similar between AW group (137 ± 294 ml) and PW group (223 ± 351 ml).

Ambient temperature, intravenous fluid administered, urine output, and volume of irrigation fluid used did not differ significantly between the two groups. Patients’ platelet counts, prothrombin time, international normalized ratio, and fibrinogen values did not differ significantly from preoperative to postoperative determinations in either group (data not shown).

### Follow-up and adverse events

Table 3 lists the postoperative outcomes (secondary endpoints) of the study. There was no difference between the two groups in LOS in the PACU, ICU or hospital. For evaluation of surgical-site infection, all patients were followed for 30 days after operation, and those who had an implant were followed for 90 days. Although three surgical-site infections were recorded in the PW group (9.4%) compared with none in the AW group, the difference is not statistically significant and is of unknown clinical significance. Shivering was recorded more often in PW patients than in AW patients, but the number of patients who had shivering was low, and difference was not statistically significant (*p* = 0.105). No cardiovascular events, including electrocardiographic abnormalities, were recorded in either patient group.

**Table 3:** Postoperative Outcomes of Patients in Two Study Groups

Variable	Passive Warming Group (*n* = 32)	Active Warming Group (*n* = 30)	*p* Value
PACU			
Patient, *N* (%)	30 (93.7)	29 (96.7)	0.2056
LOS, mean ± std (min)	35.4 ± 14.7	28.7 ± 21.8	0.0525
ICU			
Patient, *N* (%)	3 (9.4)	2 (6.6)	0.9468
LOS, mean ± std (day)	2.7 ± 1.2	7.0 ± 2.8	0.0545
Shivering, No of patients (%)	6 (18.7)	1 (3.3)	0.1048
Hospital LOS after surgery, mean ± std (day)	13.1 ± 6.2	11.1 ± 4.9	0.1989
Sugical Site Infetion, *N* (%)	3 (9.4)^a^	0 (0)	0.2384
Cardiovascular Events, *N* (%)	0 (0.0)	0 (0.0)	

*LOS* length of Stay

^a^All 3 cases of SSI were superficial incisional infection initially and then one of them evolved to sepsis

Only three patients experienced adverse events, and there was no statistically significant difference between the groups in the incidence of events (Table 4). One PW patient developed a postoperative pulmonary infection and another an anastomotic leak. One AW patient developed a pleural effusion. No severe adverse events were reported throughout the study.

**Table 4:** Adverse Events and Severe Adverse Events^a^

Variable	Passive Warming Group (*n* = 29)	Active Warming Group (*n* = 30)	*p* Value
Adverse Events, *N* (%)	2 (6.5)^b^	1 (3.3)^c^	0.5503
Severe Adverse Events, *N* (%)	0 (0.0)	0 (0.0)	

^a^based on safety data set

^b^ One case is lung infection, another case anastomotic leak

^c^One case pleural effusion

## Discussion

The adverse consequences of perioperative hypothermia have been well-established and investigated by the anesthesiology community worldwide. Besides anesthesiologists, nurses in charge of perioperative care, also have contributed to research in the area of perioperative hypothermia [13]. Maintaining normothermia is associated with enhanced recovery after surgery (ERAS) in patients, which is a multimodal, multidisciplinary approach (physicians, nurses, physician assistants, ICU workers etc.) to the care of the surgical patient [18]. Developed countries have many clinical practice guidelines and recommendations for the management of patients’ body temperature during operations, but this is not the case in China, where lack of perioperative temperature management was first appreciated in the 1990s. Recently published data [19] have shown that the incidence of perioperative hypothermia was as high as 40% in Beijing, one of the most developed urban centers in China, with the most abundant healthcare resources.

The results of this study documented that AW was associated with reduced blood loss during two kinds of major surgery: open surgical thoracic and hip replacement operations. Although this association is not proven to be cause-effect, we cannot implicate with certainty other factors than warming, and the result is consistent with other reports ^4, 5^ of increased blood loss and transfusion requirement in patients who experienced hypothermia during major operations [20]. In a recent intraoperative hypothermia survey [19], patients who underwent thoracic surgery had a higher risk of intraoperative hypothermia and lower average temperature than did those undergoing other operations. Thus, in this study, we included patients receiving intrathoracic operations. We found that the Bair-Hugger forced-air (active) warming system maintained intraoperative normothermia, even in long operations, and reduced the intraoperative hypothermia rate to nil compared with about 72% in the PW group. Although the maintenance of intraoperative normothermia was associated with significantly less intraoperative blood loss in AW patients, a statistically significant increase in transfusion requirement was not found. This apparent discrepancy may be due to the limited sample size and the wide range in transfusion volumes in both AW and PW patients. Nonetheless, the possibility that maintaining normothermia reduces the perioperative requirements of allogeneic blood and blood products is important, as it could reduce the incidence of transfusion-related infections and allergic reactions as well cost, which are important considerations in China and other countries where the supplies of donated blood are limited.

Perioperative disturbances of hemostasis are common in patients undergoing major operations, and intraoperative hypothermia can induce platelet dysfunction [21]. In this study, though, we did not find reductions in either platelet counts or other measures of coagulation (prothrombin time, international normalized ratio or fibrinogen) in the PW group. The operative time was longer in the PW patients than in the AW patients by nearly 1 h. Although this difference is not statistically significant, it raises the possibility that longer or more complicated operations was a determinant in our patients’ blood loss. Thus, the cause of perioperative blood loss may have been more complicated than hypothermia in our patients.

We acknowledge that our study has limitations: 1) The sample size is small (62 cases). 2) The nature of the medical-device trial required that it be open-label; thus, the investigators and nurses and were not blinded to the method of warming, which could have introduced bias. To help lessen possible impact of this bias we measured patients’ hemoglobin values as well as estimating blood loss through sponge weights and suction-reservoir volumes. 3) Due to small sample size, this study was underpowered to address the secondary endpoints of surgical-site infection, LOS and cardiovascular events.

In conclusion, we compared the intraoperative blood loss in patients who received major operations (open thoracic or hip replacement) and were randomized to passive warming, with a blanket, or active warming, with a forced-air warming system. Although this is a pilot study with small sample size, it was found that blood loss would be significantly more in patients treated with passive warming than in those treated with active warming. Since this was a pilot study as part of a national perioperative health-care quality improvement and health economic research program in China, a post-hoc health economic analysis and a pivotal trial-based health economic study may follow.

## Abbreviations

AE: Adverse event
ASA: American Society of Anesthesiologists
AW: Active warming
CBW: Cotton blanket warming
FAW: Forced air warning
ICU: Intensive care unit
LOS: Length of stay
OR: Operating room
PACU: Post anesthesia care unit
PT: Prothrombin time
PTT: Partial thromboplastin time
PW: Passive warming
RCT: Randomized controlled trial
SAE: Severe adverse event
SSI: Surgical site infection
